# On the Inadmissibility of the Evidence of a Lunatic in a Court of Justice

**Published:** 1851-07-01

**Authors:** 


### ON THE INADMISSIBILITY OF THE EVIDENCE OF A LUNATIC IN A COURT OF JUSTICE.

In the last number of the *Journal* we published a report of the trial of Samuel Hill for the manslaughter of a lunatic, named Moses J. Barnes, confined in Peckham House Asylum. The principal witness in the case was a man named Donelly, a patient in the same institution. The question arose as to the admissibility of his evidence. Upon this point the judges reserved their decision until Mr. Collier had fully argued the case. The matter was argued before Lord Campbell, Mr. Baron Alderson, Mr. Justice Coleridge, Mr. Baron Platt, and Mr. Justice Talfourd. Sir F. Thesiger, Mr. Clarkson, and Mr. Bodkin, appeared for the crown; Mr. Collier for the prisoner. We are fortunately able to lay before our readers Mr. Collier's argument in detail. We do this more readily, as the judges thought highly of it, and complimented Mr. Collier for the ability and learning he displayed.

The following is the case stated by Mr. Justice Coleridge for the opinion of the judges:—

This prisoner was tried before me, assisted by my brother Cresswell, at the last February sittings of the Central Criminal Court, for the manslaughter of Moses James Barnes—he was convicted; but a question was reserved for the opinion of the Court of Appeal, as to the propriety of having admitted a witness of the name of Richard Donelly, on the part of the prosecution.

The deceased and the witness were both lunatic patients in a Mr. Armstrong's asylum at Camberwell, at the time of the supposed injury, and they were at that time placed in a ward called the infirmary. It appeared that a single sane attendant (the prisoner) had the charge of this ward, in which as many as nine patients slept; and that he was assisted by three of the patients, of whom the witness Donelly was one.

It was opened for the prosecution that the witness Donelly was to be called, and, therefore, on both sides some evidence was gone into in the course of the case, and before he was called, in order to found and to meet the objection to his competency.

Muncaster, who had been an attendant in charge of the infirmary ward before the prisoner, stated thus: "Donelly labours under the delusion, that he has a number of spirits about him, which are continually talking to him; that is his only delusion; he has never been free from it, to my knowledge, since I have known him."

Joseph Stuart Burton, the medical superintendent, stated the same, but added: "I believe him to be quite capable of giving an account of any transaction that happened before his eyes. I have always found him so; it is solely with reference to the delusion about the spirits that I attribute to him being a lunatic; when I have had conversation with him on ordinary subjects, I have found him perfectly rational; but for his delusion, I have seen nothing in his conduct or demeanour in answering questions otherwise than the demeanour of a sane man."

James Hill, a doctor in medicine, who had been formerly medical superintendent at the same asylum, stated: "The memory of an insane man is not necessarily affected—it frequently is, but frequently is not. I have seen Dr. Haslam's work; I do not agree in all cases with his remark, that memory appears to be perfectly defective in all cases of insanity—certainly not; it may probably be so in the generality of cases. Madness is commonly accompanied by a great deal of excitability of the brain, but in some cases it is not; it is very often accompanied by physical irritation of the brain; it is one of the most common causes of madness, either primarily or secondarily; in certain cases of acute madness, the ideas in the mind of a madman succeed each other more rapidly than in the mind of a sane man, and in a more confused manner—that is, where there is actual irritation of the brain; it is quite possible for a man to entertain a delusion on one subject, without its affecting his mind generally on other subjects; in most cases, where a delusion prevails, and the man is mad, the rest of his mind is affected to some extent. I agree with Dr. Prichard, that 'in monomania the mind is unsound, but unsound on one point only;' there is no doubt, however, that all the mental faculties are more or less affected, but the affection is more strongly manifested in some than in others; it is difficult to ascertain without strict inquiry, the extent of a madman's delusions; they have sometimes the power of concealing their delusions even from their medical attendants, especially after having been frequently conversed with about the delusion, and knowing that they are the cause of their detention, but it is infrequent. It is a doubtful point whether what they say is not for a particular purpose—for instance, to obtain liberty. If a madman has an object to answer, he is sometimes capable of concealing his delusions. I have known it, but not as a general rule, they are probably capable of a good deal of dissimulation—many are, I know; but many do not exhibit that tendency—it is common for a certain class of madmen to exhibit a great deal of cunning. Donelly labours under a delusion with respect to spirits; he is, in the strict sense of the word, a lunatic, inasmuch as he labours under a delusion; he is not excitable by any means. I have known instances of lunatics concealing their delusions, but in all these cases there is an evident and apparent motive. I have known decided lunatics, not monomaniacs, in what are called lucid intervals, capable of going about and managing their own affairs; in ordinary cases there is no particular difference between a monomaniac, apart from his particular delusion, and an insane person in a lucid interval; during the lucid intervals of the insane person, he is all but a monomaniac—is a monomaniac all the time; in the instance of a monomaniac you produce the insanity the moment you touch the particular chord; it is possible that you might revive insanity in a madman during a lucid interval, by touching on the same subject, if it is but recent. I always found Donelly perfectly rational except on the subject of his particular delusion."

Donelly was then called, and before being sworn, was examined by the prisoner's counsel. He said: "I am fully aware I have a spirit, and twenty thousand of them—they are not all mine—I must inquire—I can where I am—I know which are mine—those ascend from my stomach and my head, and also those in my ears—I don't know how many they are—the flesh creates spirits by the palpitation of the nerves and the rheumatics; all are now in my body, and round my head; they speak to me incessantly, particularly at night. That spirits are immortal, I am taught by my religion from my childhood; no matter how faith goes, all live after my death—those that belong to me, and those that do not; Satan lives after my death, and so does the living God." After more of this kind, he added: "they speak to me instantly—*they are speaking to me now*—they are not separate from me—they are round me speaking to me now—but I can't be a spirit, for I am flesh and blood—they can go in and out through walls and places which I cannot; I go to the grave, they live hereafter, unless, indeed, I've a gift different from my father and mother; that I don't know. After death, my spirit will ascend to heaven or remain in purgatory—I can prove purgatory. I am a Roman-catholic; I attended Moorfields, Chelsea Chapel, and many other chapels round London. I believe purgatory. I am taught that in my childhood and infancy; I know what it is to take an oath—my catechism taught me from my infancy tells me when it is lawful to swear; it is when God's honour—our own or our neighbour's good require it—when man swears he does it in justifying his neighbour on a prayer-book or obligation—my ability evades me while I am speaking, for the spirit ascends to my head.

When I swear, I appeal to the Almighty—it is perjury the breaking of a lawful oath, or taking an unlawful one—he that does it will go to hell for all eternity."

He was then sworn, and gave a perfectly correct and rational account of a transaction which he reported himself to have witnessed. He was in some doubt as to the day of the week on which it took place; and on cross-examination, said: "These creatures insist upon it, it was Tuesday night, and I think it was Monday." Whereupon he was asked, "Is what you have told us what the spirits told you, or what you recollect without the spirits?" and he said, "No, the spirits assist me in speaking of the date; I thought it was Monday, and they told me it was Christmas eve, Tuesday; but I was an eye-witness, an ocular witness, to the fall to the ground."

The question for the opinion of the Court is, whether this witness was competent. Sentence has not been passed, but is postponed until this question has been decided, and the prisoner remains in custody.

Mr. Collier's argument was as follows:—I. Donelly was, both at the time of the occurrence to which he spoke and of the trial, *non compos mentis*, in the legal, medical, and ordinary sense of the term. He was an inmate of a lunatic asylum, into which he could not have been legally admitted without two medical certificates of his being insane, and a fit person to be confined; or, if he were a pauper, without an order of justices adjudicating these facts.* And if he had been restored to reason, he must have been discharged.† He was declared by one of the medical witnesses to be, "in the strict sense of the term, a lunatic," labouring under an insane delusion, from which he was never free. The characteristic symptoms of insanity are declared by Dr. Willis to be, "a confirmed belief in an assumed idea, upon which the patient is always acting, without any apparent bodily disease, to the truth of which he would pertinaciously adhere, in opposition to the plainest evidence of its falsity."‡ And this view is adopted by Sir John Nicholl, in the case of Dew *v.* Clarke,§ who says, "The true criterion, the true test, of the absence or presence of insanity, I take to be the absence or presence of what, used in a *certain* sense of it, is comprisable in a single term,—namely, *delusion*. Wherever the patient once conceives something extravagant to exist, which has, still, no existence whatever but in his own heated imagination; and wherever, at the same time, having once so conceived, he is incapable of being, or, at least, of being *permanently*, reasoned out of that conception; such a patient is said to be under a *delusion*, in a particular, *half technical*, sense of the term; and the absence or presence of delusion, so understood, forms, in my judgment, the true and only test, or criterion, of absent or present insanity. In short, I look upon delusion, in this sense of it, and insanity, to be almost, if not altogether, convertible terms; so that a patient under a delusion, so understood, on any subject or subjects, in any degree, is, for that reason, essentially mad, or insane, on such subject or subjects, in that degree. On the contrary, in the absence of any such delusion, with whatever extravagances a supposed lunatic may be justly chargeable, and how like soever to a real madman he may either speak or act on some or on all subjects, still, in the absence, I repeat, of anything in the nature of *delu-*

* See 8 and 9 Vic. c. 100, § 45, and 8 and 9 Vic. c. 126, § 51.
† See 8 and 9 Vic. c. 100, § 76, 77.
‡ Willis on "Mental Derangement," pp. 20, 21.
§ 3 Add. 90.

*sion*, so understood as above, the *supposed* lunatic is, in my judgment, not properly or essentially insane."

And Sir John Nicholl proceeds to cite, in confirmation of this view, a treatise by Dr. Battie, as well as the treatise by Dr. Willis, above quoted. Sir J. Nicholl further discusses the question of partial insanity, on which subject his decision has been reviewed by Lord Lyndhurst* in these terms:—"I collect that his meaning is this: that there must be unsoundness of mind in order to invalidate a will, but that the unsoundness may be evidenced in reference to one or more subjects. It seldom happens, he says, that a person who is insane displays that insanity with reference to every question and every subject: it shows itself with reference to particular subjects, and sometimes with reference to only one individual subject. It sometimes displays itself with reference to one subject very decidedly, and very generally, perhaps, with reference to other subjects. All that the learned judge meant to convey was, that it was no objection to the imputation of unsoundness that it manifested itself only, or principally, with reference to one particular question or one particular person; and he illustrates his position by a number of cases, some of them of public notoriety, and known to us all. This construction does not rest on any general reasoning, because, for the purpose of avoiding misapprehension, and as if his attention had been directed to the very point, he himself, in the course of his judgment, explains in distinct terms what he meant by the term *partial insanity*. 'It was said,' he observes, 'that partial insanity was unknown to the law.' The observation could only have arisen from mistaking the sense in which the court had used that term. It was not meant that a person could be partially insane and sane at the same moment of time. To be sane, the mind must be perfectly sound, otherwise it is unsound. All that was meant was, that the delusion may exist only on one or more particular subjects. In that sense, the same term is used by no less an authority than Lord Hale."† This view of Lord Lyndhurst's is said to have been adopted by the medical profession in Dr. Taylor's work on "Medical Jurisprudence," p. 627, 1st edit., in which it is said, "The only admissible view of this disorder (monomania) is that which was taken by Lord Lyndhurst in one of his judgments. In monomania, the mind is unsound; not unsound in one point only, and sound in all other respects; but this unsoundness manifests itself principally with reference to some particular object or person." And the authority of Dr. Prichard is also quoted. In Dr. Guy's "Medical Jurisprudence," mania is classed as one of the divisions of unsoundness of mind, and monomania as a subdivision of mania. These authorities have been cited to show that, assuming Donelly to be a monomaniac, it is incorrect to say that his mind is partly sound and partly unsound—a term applicable to the body, which consists of parts, but not to the mind, which is one and indivisible—but that his mind must be considered as diseased.

Mr. Baron Alderson—"Is every delusion a proof of madness?"

Mr. Collier—Every delusion, as explained by Dr. Willis. There may

* Dew *v.* Clarke, 5 Russ. 166, 168.

† "There is a partial insanity of mind, and a total insanity. The former is either in respect to things, (*quoad hoc, vel quoad illud insanire*—some persons that have a competent use of reason, in respect to some subjects, are yet under a particular *dementia* in respect of some particular discourses, subjects, or applications); or else it is partial in respect of degrees, (and this is the condition of very many, especially melancholy persons, who, for the most part, discover their defect in excessive fears and griefs, and yet are not wholly destitute of the use of reason)."—Pleas of the Crown, part 1, c. 4, p. 29. See also the Judgment of Lord Brougham in Waring *v.* Waring, 6 Moore's Privy Council Reports, in which the same doctrine is affirmed.

be delusions of the senses without insanity: the eye and ear may be deceived by spectres and imaginary voices; but if the patient is aware of the delusion, or capable of being persuaded of it, he is not mad. Nor is mere false reasoning, to whatever absurdities it may lead, necessarily a proof of madness. Locke says, that madmen generally reason correctly, but their premises are false. An insane delusion is a false impression concerning some matter of fact, which is constantly present to the mind, and out of which it is impossible to reason the patient. Any such delusion shows the existence of disease, the extent of the delusion indicating its virulence.

Nor was Donelly, at the time of the occurrence or of the trial, in a lucid interval, which means an interval of complete sanity, or freedom from disease. On this subject, Sir John Nicholl says, in the case of Wheeler *v.* Alderson,* "I am not able exactly to understand what is meant by a lucid interval, if it does not take place when no symptom of delusion can be called forth at the time. How but by the manifestation of the delusion is the insanity proved to exist at any one time? The disorder may not be permanently eradicated; it may only intermit. It may be liable to return; but if the mind is apparently rational on all subjects, and no symptom of delusion can be called forth on any subject, the disorder is for that time absent. There is then a lucid interval, if there be such a thing as a lucid interval, because it is difficult to ascertain the total absence of all delusion." And this is the view taken by Dr. Haslam:†—"I should define a lucid interval to be a complete recovery of the patient's intellects, ascertained by repeated examinations of his conversation, and by constant observation of his conduct, for a time sufficient to enable the superintendent to form a correct judgment. Unthinking people are frequently led to conclude that if, during a short conversation, a person under confinement shall betray nothing absurd or incorrect, he is well, and often remonstrate on the injustice of secluding him from the world. Insane people will often, for a short time, conduct themselves, both in conversation and behaviour, with such propriety, that they appear to have the just exercise and direction of their faculties; but let the examiner protract the discourse until the favourite subject shall have got afloat in the madman's brain, and he will be convinced of the hastiness of his decision."

Observations to the same effect are made by Dr. Prichard and other medical authorities.

Now, however rationally Donelly may have conversed on any subject, the medical evidence and his own account of himself, demonstrated that the delusion always existed, capable of being called forth by any allusion to it: he was therefore one of that species of *non compotes mentis* (a generic term approved of by Lord Coke),‡ known to medical as well as legal writers, and popularly as a lunatic without lucid intervals.

II.—The authorities are uniform, that as a general proposition a person *non compos mentis* cannot be examined as a witness, and no qualification is engrafted upon this proposition by any text writer.

In "Comyns' Digest," Testamoigne Witness, A. 1., "who shall not be a witness," four heads are enumerated—1st, non compos; 2nd, infidel; 3rd, person convicted of treason or felony; 4th, any infamous man, and interested witnesses might have been added before Lord Denman's Act.

All these heads of objection to a witness may be resolved into two:—
1st. That he does not know the truth.

* 3 Hagg. Ecc. Report, 599.

† Haslam on Madness, 46, 47.

‡ Co. Litt. 247 (a).

2nd. That he cannot be depended upon to tell it.

The *non compos* is supposed to have an understanding, either so imperfectly developed, or so diseased, as not to be *able* to give credible testimony.

The infidel, though able to give credible testimony, is supposed to be without a sufficient motive for doing so.

So, convicted or infamous persons are supposed to have an imperfect sense of the moral and religious obligation of an oath.

Interested persons, before Lord Denman's Act, and even now, parties to the suit, are supposed to have too strong a temptation to deceive, for them to be depended upon to tell the truth.

Under the first head, *non compos*, Chief Baron Comyns says, "every witness must be credible, and therefore a man of non-sane memory shall not be allowed as a witness, as an idiot, a lunatic during his lunacy; so one within age of discretion, so an infant who does not know the nature of an oath, but a lunatic may be a witness in *lucidis intervallis*.

Mr. Baron Alderson—"Is not the test of a lunatic's competency the same as that of a child, viz., whether or not he understands the nature of an oath?"

Mr. Collier—That test does not apply to a lunatic. It may be fairly assumed that when the intellect of a child is sufficiently developed to apprehend abstract ideas, such as those of right and wrong, the existence of a God and an unseen world, his perceptions are sufficiently accurate, and his memory sufficiently retentive to enable him to know the truth respecting matters which he has seen or heard: nor is there reason for supposing him less capable of giving evidence on one subject than another; a child whose intellect is so far developed, is therefore reasonably considered *compos mentis;* but the lunatic is confessedly *non compos*, on one subject, if not more, his perceptions or imagination being false; he therefore, on one subject at least, cannot know the truth.

Religious sentiment is compatible with the most morbid imaginations; the lunatic may know the nature of an oath, and yet believe himself a King, the Pope, or the Devil, and may be ready to swear that he is each or all of these. The test which applies to a sane intellect in the course of development, is not necessarily applicable to an adult intellect diseased; accordingly, it is not said that "a lunatic shall be inadmissible who does not understand the nature of an oath," but generally that "a lunatic is inadmissible," except in a lucid interval, when he is (correctly speaking) no lunatic.

It is true that a child ignorant of the nature of an oath would be disqualified from want of religious knowledge, but this ground of disqualification is common to all persons, and the child's knowledge of an oath is considered by Comyns, not with reference to his being "an infidel," but with reference to his being "non compos." In short, a child ignorant of an oath, and a lunatic ignorant of an oath, are both doubly disqualified, *i. e.*, from want of understanding and want of religion; the absence of one of these disqualifications in a child proves the absence of the other: but not so in a lunatic. Although, therefore, the converse of the proposition, "a child ignorant of the nature of an oath is inadmissible" holds good, the converse of the same proposition with regard to a lunatic, does not. The expression, a religious lunatic, involves no inconceivable idea, but a sane child, capable of religious knowledge and no other, is barely conceivable.

In Buller's Nisi Prius, the same division is made of the heads of disqualification, which are: 1st, want of integrity; 2nd, want of discernment. The former head is said to comprise five classes of persons: 1st, persons interested; 2nd, persons stigmatized; 3rd, infidels; 4th, persons excommunicated; 5th, popish [recusants. The second head is said to comprise: 1st, idiots; 2nd, madmen; 3rd, children. With respect to the first two, no exception is laid down; but it is said, with regard to children, "There seems to be no precise time fixed wherein they are excluded from giving evidence; but it will depend in a great measure on the sense and understanding of the child, as it shall appear on examination by the court." In Co. Litt* it is said if a witness be an infidel, or of non sane memory (subsequently explained as *non compos*†), or not of discretion, or a party interested, or the like, he cannot be a witness. No case is reported in which it has been directly decided that a lunatic is not admissible; but there are several in which this has been assumed to be a settled maxim of law.

In Reg. *v.* Eriswell,‡ a question arose whether what had been said by a pauper who had become insane, relative to his settlement, was admissible. The case stated, in general terms, "the pauper continued insane at the time of hearing this appeal;" upon this Buller, J., says, "Before I state the grounds on which I hold the statement admissible, I think proper to premise that I consider the pauper as dead: he being in such a state as renders it impossible to examine him;" and Lord Kenyon, who differed with Buller, J., in one point, says, "I admit that this man, who was proved to be insane, is to be considered, as to this purpose, in the same state as if he were dead; and, it has been decided that, in such cases, the party's handwriting may be proved as if he were actually dead."

In Currie *v.* Child§, on its being shown that the attesting witness to a note was insane, Lord Ellenborough held that evidence of his handwriting was sufficient to prove the making of the note; and in Bennett *v.* Taylor,|| the Lord Chancellor allowed evidence to be given of the handwriting of an insane witness to the codicil of a will.

Professor Alison thus states the law of Scotland on this subject.¶ "It results from the first principles of evidence that no person is to be allowed to give testimony who is not fully aware of the import of what he is swearing, and capable of fully understanding the facts involved in his deposition. Of course idiots, madmen, or lunatics, must be excluded if they are either constantly in that condition, or subject to such frequent returns of the malady, and at such short intervals, as renders their testimony unfit to be relied on. If one be deranged at times only, his testimony may be taken at least *cum notâ*, concerning any matter which has fallen under his observation, when he is in a state of sound health, if he is in the same state when examined at the trial, and no such serious fit of insanity has since intervened as to cloud his recollections, and cause him to mistake the illusions of his imagination for the events he has witnessed; but if these requisites be awanting, he should either be totally rejected, or received with the greatest caution. The law of England is the same on this head."

An Irish text writer says**—"We have already seen that an *idiot* is one who from his nativity by a perpetual infirmity is *non compos mentis*, and must therefore be utterly incapable of giving evidence. But *lunatics* who are capable of enjoying intervals of sound mind, may, during those lucid

* 6, B. † 247 a. ‡ 3 T. R. 707.

§ 3 Campb. 282. See Chapman *v.* Graves, 2 Campb. 333, n.; Adams *v.* Ker, 1 Bos. and P. 360; Cunliffe *v.* Sefton, 2 East, 183.

|| 9 Ves. 381.

¶ Alison's "Practice of the Criminal Law of Scotland," p. 435, book XIII., § 395.

** Gabbett's "Criminal Law," vol. 2, p. 473, book 2, c. 14, "of the evidence," tit. 1. "Incompetency arising from *want of understanding*."

intervals, be admitted as witnesses, if they have sufficiently recovered their understanding. It should, however, appear before any such person is received as a witness, that he was in possession of his reason at the time of the event to which he testifies, as well as at the time of his examination; and that no serious fit of insanity has intervened, so as to cloud his recollection, and cause him to mistake the illusions of imagination for the facts to which he testifies."

The same rule prevails in both the civil and the canon law, and is thus stated in *Mascardus de Probationibus.** *Conclusio,* 828, p. 373.

1. Furiosus testis esse non potest.
2. Furiosus pro mortuo et absente habetur.
3. Freneticus testis esse non potest.
4. Indiscretus testis esse non potest.

Lord Campbell—"The civil law which has been said to be the perfection of reason, showed itself by no means reasonable in rejecting witnesses on many frivolous grounds."

Mr. Collier—"Probably indiscretus here is used in the sense *non compos.* as in Co. Lit. 6. b.—Mascardus proceeds—

5. Furiosus habens dilucida intervalla testis esse non potest.
6. Furiosus, nunc suæ mentis compos, potest testificari de iis, quæ vidit dum esset in furore.
7. Furiosus adhuc furens non potest testificari de iis, quæ vidit tempore sanæ mentis.

Grotius de jure belli ac pacis† treating de jure jurando, says, "primum quod de promissis et de contractibus diximus, et hic habet locum, ut animus rationis compos requiratur."

And in Heinnecius ad Pandectas, there is a passage to the same effect.

The general proposition of law that a non compos is inadmissible as a witness being thus established, there seems to be no reported case or dictum by which it is in any way qualified.

Baron Parke has indeed referred the court to a case in which he admitted a witness, proved to be, to a certain extent, insane; and on referring the question to the judges, they were of opinion that the witness was rightly admitted. This case, however, was not argued, nor was any judgment pronounced.‡ Sir David Dundas has also a note of a case in which Baron Hullock admitted, as a witness, a surgeon who had been acquitted of murder on the ground of insanity, and was then in confinement.

Mr. Baron Alderson.—"I defended that surgeon—he was no more mad than you. He practised extensively when in prison."

Mr. Collier—Undoubtedly a verdict of acquittal, on the ground of insanity, would be a very precarious test.

III. It would be inconvenient, as well upon grounds of public policy as upon other grounds, to introduce a modification of this rule. Unquestionably, the generality of the rule which exempts a lunatic from responsibility for criminal acts, has been modified: and the question in each case has been said to be, whether or not he was able to distinguish right from wrong with reference to the criminal act. But the exemption from responsibility for crimes is founded upon a sense of the injustice of punishing a person for doing that which he does not know to be wrong: a totally different foundation from that of the rule which excludes a lunatic from being a witness—an exception to the one is not, therefore, necessarily an exception to the other. On this subject Lord Erskine, in his speech for Hatfield, says§ :—

"There is a wide distinction between civil and criminal cases. If, in the

* p. 373. † Lib. 2, c. 13, § 2. ‡ Rex *v.* Morley. § State Trials, vol. 27, p. 1311.

former, a man appears, on the evidence, to be *non compos mentis*, the law avoids his act, though it cannot be traced or connected with the morbid imagination which constitutes his disease, and which may be extremely partial in its influence on his conduct; but, to deliver a man from responsibility for crimes, I am by no means prepared to apply this rule, however well established, when property only is concerned. In the very recent case of Mr. Greenwood, which must be fresh in his lordship's recollection, the rule in civil cases was considered to be settled. That gentleman, while insane, took up an idea that a most affectionate brother had administered poison to him. Indeed, it was the prominent feature of his insanity. In a few months he recovered his senses. He returned to his profession as an advocate; was sound and eminent in his practice, and in all respects a most intelligent and useful member of society; but he could never dislodge from his mind the morbid delusion which disturbed it. The noble and learned judge who presides at this trial, and presided at that, told the jury that if they believed Mr. Greenwood, when he made his will, to be insane, the will could not be supported, whether it had disinherited his brother or not; that the act, no doubt, strongly confirmed the existence of the false idea which, if believed by the jury to amount to madness, would equally have affected his testament, if the brother, instead of being disinherited, had been in his grave, and that, on the other hand, if the unfounded notion did not amount to madness, its influence could not vacate the devise. This principle of law appears to be sound and reasonable, as it applies to civil cases, from the extreme difficulty of tracing, with precision, the secret motives of the mind, deprived by disease of its soundness and strength. Whenever, therefore, a person may be considered *non compos*, all his civil acts are void, whether they can be referred or not to the morbid impulse of his malady, or even though, to all visible appearances, totally separated from it."

And this doctrine has been confirmed in the case before cited, of Dew *v.* Clarke.*

It has also been laid down, generally, that a lunatic is incapable of filling any office, of being a member of parliament, trustee, executor, &c., and his liability on contracts has been limited to those which relate to necessaries supplied to himself, contracts which, therefore, must be invariably for his benefit.

It is impossible for the court to say that all lunatics are admissible, and that their credit is a question for the jury;——

[Lord Campbell—Certainly.]

—if so, what degree of lunacy shall be accounted such as not to disqualify? Shall delusions on one subject, or two, or upon several, and if so, upon how many, be held compatible with credibility? Again, it is by no means easy to say what is *one* subject with reference to insane imaginations. This difficulty is illustrated by the present case; where, though the medical men described the delusion as extending only to one subject, viz., converse with spirits, it turned out that the lunatic believed the spirits to converse with him on every subject which happened to arise. It must always be a work of extreme difficulty to define the limits of an insane delusion, to trace its ramifications through the mind, and to pronounce that it has not more or less vitiated and falsified all the faculties.

But it may be said that the question is, whether or not the delusion relates to the subject matter of the trial. This, however, is a matter into which it is impossible to inquire: the judge cannot know beforehand what evidence will or will not be material at the trial. Even assuming him to

* See *Ante*, and see Warner *v.* Warner, *Ante.*

have collected from the depositions the probable aspect of the case for the prosecution, it is impossible for him to know that of the defence; nor, in civil cases, can he anticipate the aspect of the case on either side; he cannot, therefore, say that the subject of the insane delusion may not materially affect the trial.

And herein consists the difference which makes it so much more difficult to ascertain a man's lunacy with reference to his capacity for being a witness, than to determine it with reference to his capacity to perform an act — such as to make a will, or his responsibility for a crime. In the two latter cases, his lunacy is inquired into with reference to a *past* transaction which is known, in the former with reference to something which he is to do, not ascertainable.

Lord Campbell—There always must be an inquiry with reference to the state of a witness's mind.

Mr. Collier—Unquestionably; but when the inquiry has ascertained the fact of his being a lunatic, it is more convenient that it should end there, than that the judge should proceed to investigate whether or not the lunacy is likely to affect something which he cannot know, viz., the evidence which the witness is to give at the trial. Whether or not a witness's mind is unsound, will, in most cases, be ascertainable with no great difficulty, whereas a further inquiry into the nature and extent of the unsoundness, what faculties of the mind it may or may not affect, and to what subjects it may relate, must always be a task of great difficulty, involving a necessarily painful examination in public of the lunatic himself, possibly attended with the consequences of aggravating his malady, and always unsatisfactory, because it is impossible to test his insanity with reference to every subject which may arise at the trial. In the present case, the medical evidence clearly proved the lunacy of the witness, before he was called, and if the rule contended for had been adopted, he would have been spared the examination on the subject of his delusions, to which he was necessarily subjected.

Again; if a lunatic witness be admitted on either side, and his credibility left to the jury, the other side must be permitted to call any number of witnesses to prove the extent of his lunacy, to be contradicted possibly by witnesses to his comparative sanity. It is true that juries have always to decide on the credibility of witnesses, but their decision on this rests on the demeanour of the witnesses and the probability of the facts deposed to; nor are witnesses allowed to be called as to the character, habits, or modes of thought of another witness, or asked a question as to his credibility beyond this, whether or not they would believe him on his oath. Whereas a conflict of witnesses, as to the extent and nature of the insanity of another witness, would involve the jury in a very complicated collateral question, which, when it is *the* question in issue (as in trials relating to the validity of wills and some criminal cases), they find it sufficiently difficult to determine.

IV.—Lastly, assuming that the generality of the rule should be qualified in any cases, the present case does not fall within any admissible qualification of it.

Here the lunatic believed himself at the time of the trial, and frequently, in converse with spirits, who proceeded from his stomach, and sat in his ears, while he was occasionally visited by the spirit of the Queen, and of Luther, and others, which he called controversial spirits. These spirits spoke to him on the subject of the trial, and differed from him as to the date of the injuries inflicted upon Barnes; a fact material to the inquiry, because part of the evidence against the prisoner was, that several days had elapsed between the commission of the injuries and his communicating them to the medical officer of the asylum, during which it was assumed that he must have become cognizant of them, and would have reported them if he had not been the party who inflicted them. Under these circumstances, it is submitted that there would be no probability of Donelly being convicted of perjury, if any part of his evidence was false, and that although he gave answers indicating some notion of the nature of an oath in the abstract, he was practically not subject to the penalties of perjury—a protection to which the party deposed against is always entitled, —and was not admissible.

Lord Campbell, C.J.—I am glad this case has been reserved, for the matter is of great importance, and ought to be decided. However, after a very learned argument, which I have heard with a great deal of pleasure, I entertain no doubt that the rule is as was laid down by Parke, B. in the unreported case that has been referred to, that, whenever a delusion of an insane character exists in any person who is called as a witness, it is for the judge to determine whether the person so called have a sufficient sense of religion in his mind, and sufficient understanding of the nature of an oath, for the jury to decide what amount of credit they will give to his testimony. Various authorities have been referred to, which lay down the law, that a person *non compos mentis* is not an admissible witness. But in what sense is the term *non compos mentis* employed? If a person be so to such an extent as not to understand the nature of an oath, he is not admissible. But a person subject to a considerable amount of insane delusion may yet be under the sanction of an oath, and capable of giving very material evidence upon the subject matter under consideration. The just investigation of the truth requires such a course as has been pointed out to be pursued, and in the particular circumstances of this case I should have adopted the course which was taken at the trial; nothing could be stronger than the language of the medical witnesses in this case to show that the lunatic might safely be admitted as a witness. It has been contended that the evidence of every monomaniac must be rejected. But that rule would be found at times very inconvenient for the innocent as well as the guilty. The proper test must always be, does the lunatic understand what he is saying, and does he understand the obligation of an oath? The lunatic may be examined himself, that his state of mind may be discovered, and witnesses may be adduced to show in what state of sanity, or insanity, he actually is; still, if he can stand the test proposed, the jury must determine all the rest. In a lunatic asylum the patients are often the only witnesses to outrages upon themselves and others, and there would be impunity for offences committed in such places, if the only persons who could give information were not to be heard.

Alderson, B.—I quite agree that it is for the judge to say whether the person called as a witness understands the sanction of an oath, and for the jury to say whether they believe his evidence. Here the account of the lunatic himself, and the evidence of the medical witnesses. show that he was properly received as a witness.

Colleridge, J.—This is an important case. We have been furnished during the argument with rules dr[illegible] the older authorities against the admissibility of a lunatic witness, which are stated without any qualification. It was not necessary for the decision of those cases that the rule should be qualified, and in former times the question of competency was considered upon much narrower grounds than it is at present. The evidence in this case left the matter thus: there was a disease of the mind of the witness, operating upon particular subjects, of which the transaction of which he came to speak was not one. He was perfectly sane upon all other things than the particular subject of his delusion. As far as memory was concerned, he was in the position of ordinary persons, and upon religious matters he was remarkably well instructed, so as to understand, perfectly, the nature and obligation of an oath. If it had appeared, upon his evidence, that his impressions of external objects were so tainted by his delusion that they could not be acted upon, that would have been a ground for the jury to reject, or give little effect to his evidence. But this was a matter for them to determine.

Platt, B. concurred.

Talfourd, J.—If the proposition that a person suffering under an insane delusion cannot be a witness were maintained to the fullest extent, every man subject to the most innocent unreal fancy would be excluded. Martin Luther believed that he had had a personal conflict with the devil; Dr. Johnson was persuaded that he had heard his mother speak to him after death. In every case the judge must determine according to the circumstances and extent of the delusion; unless judgment and discrimination be applied to each particular case, there may be the most disastrous consequences.

Conviction affirmed.

---

